# Trade Unionism and the Medical Profession

**Published:** 1919-03-01

**Authors:** 


### TRADE UNIONISM AND THE MEDICAL PROFESSION.

As on previous occasions of a kindred order all the speakers at the Wigmore Hall meeting of the 23rd ult. agreed that if medical opinion is to speak effectively in relation both to its own values and to the health needs of the community the first necessity of the profession is organisation and union. Further, it was unanimously allowed that to be of service the union of the profession must be achieved without delay. Existing grievances of the practitioner, suffered at the hands of the Insurance Commissioners and other Government departments, were eloquently exposed, and the prospect that similar conditions are likely to be established under the Ministry of Health was emphasised. Yet though for these two pressing considerations urgency was claimed the leading speakers announced that no possible remedy existed outside the establishment of a medical trades union. This was the one refuge offered from the further invasion of the rights of the profession by a tyrannical, overbearing, and interfering bureaucracy. If this be so it must at once be admitted that the prospect is not a happy one. For, whether rightly or wrongly, it is certain that the great majority of the profession are opposed to the adoption of trades union methods, and even the most enthusiastic advocate of these methods cannot hope to convert some thousands of his *confrères* in time to speak with authority on legislative proposals now actually before Parliament. If reason and argument and a claim for fair dealing are out of date resources and a trades union the only reliable weapon, then the profession may abandon its cause in despair, for most certainly much water will flow under London Bridge before this weapon will be accepted by the majority of practitioners. Whatever value the new policy may have it has on the present occasion one serious defect. It will not be in time for the argument, for it can only arrive a day behind the fair.

There can, of course, be no challenge to the claim that the medical profession has the right to decide the terms on which its members shall give their co-operation to any form of organised Government service, and the machinery for making this claim effective already exists. All that is required is the determination to utilise it. To thrust this aside and to try to set up a new organisation on a trades union basis will, however excellent may be the intentions of the proposers, merely accentuate division, for it is notorious that opposition to such an aim is deep-rooted in the profession. In the circumstances of the moment the attempt is prejudicial to the unity which its advocates desire to serve, and in view of the actual situation the proposal can hardly be regarded as a serious one.

The whole of the argument relative to trades union methods in medicine involves many considerations having legal, ethical, and traditional values. Upon these we for the present say nothing. Our desire is to deal with the position as it exists here and now. We agree that an authoritative and effective organ of professional opinion is much to be desired, not less in the interests of the community than in the interests of the profession itself. But we are quite sure that to ask the profession to agree to found its organisation on a trades union basis will prove an opportunity not for united action but for controversy and division.

